# The validation of a Japanese language version of the postoperative quality of recovery scale: a prospective observational study

**DOI:** 10.1186/s40981-021-00432-0

**Published:** 2021-04-09

**Authors:** Koki Yamashita, Stuart Boggett, Yoshifumi Kodama, Isao Tsuneyoshi, Colin Royse

**Affiliations:** 1 Department of Anesthesiology and Intensive Care, Faculty of Medicine, University of Miyazaki, Miyazaki, Japan; 2 Department of Surgery, The University of Melbourne, Room 214, Level 2, Alan Gilbert Building 161 Barry St, Parkville, Melbourne, Victoria 3010 Australia; 3 Australian Director, Outcome Research Consortium, Cleveland Clinic, Cleveland, OH USA

**Keywords:** Postoperative quality of recovery, Validation, Feasibility, Bilingual translation

## Abstract

**Background:**

The Postoperative Quality of Recovery Scale (PostopQRS) is a survey-based tool that measures quality of the postoperative recovery in multiple domains over multiple time periods. The purpose of this study is to validate the Japanese version of the PostopQRS.

**Methods:**

A prospective observational study using bilingual healthy volunteers was conducted in Australia to assess equivalence of the test values between the two languages. To assess the feasibility and discriminant validity of the PostopQRS in a Japanese population, an observational study was conducted on patients undergoing ear-nose-throat and orthopedic surgery in Japan, with measurements performed prior to surgery, 2 h, and 1, 3, and 7 days following surgery. The survey was conducted face-to-face while in hospital and via the telephone following discharge.

**Results:**

Sixty-eight volunteers participated in the validation study. The scores in the Japanese version were similar to the English version in all domains at all timepoints. In the cognitive domain, there were no differences between the Japanese and English versions for word recall and word generation tasks. For digits forwards and digits backwards the values were skewed to the maximal value, and although significantly different, the absolute difference was <10% at all timepoints between English and Japanese versions. Fifty-one patients, ear-nose-throat (*n*=22) and orthopedic (*n*=29), were included in the clinical study. Orthopedic patients had a significantly worse recovery profile over time in overall recovery (*p*<0.01), physiological (*p*=0.02), nociceptive (*p*=0.03), and activities of daily living (ADL, *p*<0.01) domains, but was not different for emotive (*p*=0.30) or cognitive domains (*p*=0.10).

**Conclusion:**

The Japanese version of the PostopQRS is similar to the English version and was able to discriminate recovery between different surgery disciplines.

**Trial registration:**

UMIN, UMIN000033268, Registered 6 August 2018.

**Supplementary Information:**

The online version contains supplementary material available at 10.1186/s40981-021-00432-0.

## Introduction

The measurement of postoperative quality of recovery over multiple time periods is integral to improving recovery after surgery [1]. The Postoperative Quality of Recovery Scale (PostopQRS) developed by Royse et al. [2] is a tool that measures quality of recovery in five domains (physiological, nociceptive, emotive, activities of daily living (ADL), cognitive function) and one subjective domain based on the patient perspective (see www.postopqrs.com). It is a verbal scale that has been validated for both face-to-face as well as telephone conduct [3]. The assessment tool has been used to identify differences in the quality of recovery when comparing different operative procedures [4], anesthesia techniques [5], and therapeutic interventions [6]. It has also been extensively validated, including two modifications to the cognitive domain, in which a tolerance factor was included due to performance variability observed in human volunteers not undergoing surgery [3], and a method was introduced to score cognitive recovery in patients with low baseline cognition [7].

The PostopQRS has been translated into Spanish, French, Portuguese, German, Mandarin, Arabic, Japanese, and is in the process of undergoing translation into Malay, Korean, Greek, Swedish, Yugoslavian, and Russian. Validation studies have been separately published for the Chinese [8], Arabic [9], and Portuguese [10] versions. A Japanese version has been previously translated and published as a pilot study but had not considered the differences in the language styles within the word generation task of the cognitive domain in detail [11].

The cognitive domain is the most likely to be affected by language translation due to the potential for different usage of words within cultures and frequency of words that originate with particular letters of the alphabet. In the Japanese language, there is cursive syllabary called “Hiragana,” which has similarities to the English alphabet. Each character has a sound made up of a combination of a vowel and a consonant that can be written in English. In this study, we used the Hiragana characters; “う (u),” “か (ka),” and “せ (se)” to replace the English letters in the word generation task.

Validation of a new language version requires two separate studies. To test equivalence of the language versions, the PostopQRS must be conducted in both languages over multiple time periods in healthy bilingual volunteers who are not undergoing surgery. This tests stability of the scale over time, as the scores should vary little over the time period. The scale must also be conducted in the population being studied to assess feasibility, acceptability, and discriminant validity, using at least two cohorts undergoing surgery with known differences in their recovery profile.

The aim of the study was to evaluate the validity of the Japanese version of the PostopQRS for equivalence with the English version, and to test the use of the scale in Japanese surgical cohorts.

## Methods

The prospective observational studies were approved by the Human Ethics committee of The University of Melbourne (1544471.2) and the Ethics committee of The Miyazaki University Hospital (O-0366). The study conforms to the ethical guidelines of the Declaration of Helsinki and written informed consent was obtained from all participants. The clinical study was registered prior to any participant enrollment, on the UMIN Clinical Trial Registry, https://upload.umin.ac.jp/cgi-open-bin/ctr_e/ctr_view.cgi?recptno=R000036903 (UMIN000033268, Principal investigator: Prof Colin Royse, Date of registration: 1 August 2018). The manuscript adheres to the applicable STROBE guidelines [12].

### Trial design

The original PostopQRS version (Supplemental file 1) was both forward and backward translated by specialist translators. The revision of the Japanese PostopQRS version was amended by two bilingual but native Japanese speakers. There were two steps involved in the validation of the Japanese version of the PostopQRS. Firstly, to ensure the psychometric properties of the Japanese questionnaire, a prospective observational study using bilingual healthy volunteers was conducted from May to August 2018 in Australia; they were recruited via advertisement on social media platforms. Written informed consent from volunteers was obtained either in person or via mail. The PostopQRS assessment was completed either in person or over the phone for all timepoints measured in the study.

Secondly, to assess the discriminant validity of the tool in a Japanese population, a prospective observational study was conducted on patients undergoing ear-nose-throat (ENT) and orthopedic surgery between August 2018 and January 2019 in Japan.

Exclusion criteria for both studies were age <18 years old and participants with inadequate cognitive ability to complete the PostopQRS survey or provide consent. For the clinical study, patients who were considered at high risk for postoperative ventilation were excluded.

### Data collection

For the volunteer study, participants completed all sections of both the English and Japanese versions of the PostopQRS, in the nociceptive, emotional, activities of daily living (ADL), and cognitive domains over four timepoints; at baseline, and days 1, 3, and 7. In the first step, we used three parallel forms in the cognitive domain to reduce the learning effect. These parallel forms included different numbers, words, and letters for each task. Moreover, we randomized the order of the language and the three parallel forms used at each time point.

For the assessment of discriminant validity, the Japanese version was conducted on five occasions; at baseline prior to surgery, 2 h, and days 1, 3, and 7 after the procedure. The physiological domain was assessed only at baseline, 2 h, and days 1 and 3. The ADL domain was completed at baseline, and days 1, 3, and 7. For both parts of the study, the initial baseline testing was performed face-to-face and subsequent testing was performed either face-to-face or via telephone according to the participant’s preference. To assess the feasibility, acceptability, and discriminant validity of the Japanese version of the PostopQRS (Supplemental file 2), we conducted a prospective observational study for clinical patients at the Miyazaki University Hospital. Only the Japanese version of the PostopQRS was used for the clinical study. Surgical specialties were ENT and orthopedic surgery; both procedures included only elective cases. For the ENT group, we investigated typical types of ENT procedures, such as intraoral surgery (e.g., tonsillectomy), nasal surgery (e.g., deviatomy), and ear surgery (e.g., tympanoplasty). Orthopedic procedures included lower extremity surgery, for example, total hip replacement (THR) and total knee replacement (TKR). The anesthetic technique was dependent on the anesthetists. After surgery, patients were directly taken to the ward from the operating theater, where the PostopQRS assessments were conducted accordingly. In all cases, we measured the level of satisfaction on how the PostopQRS assessment was conducted by asking the participants close-ended questions following the completion of each PostopQRS assessment (Supplemental table 1). Major complications and date of discharge were also recorded. For the calculation of the recovery scores, we followed the same rules published previously for the PostopQRS [2, 3, 7].

### Statistical analysis

We described data as median (IQR) for continuous variables, and absolute number (%) for categorical variables. In the bilingual volunteer study, the data of each task was compared between Japanese and English versions using Kruskal-Wallis *H* test the non-parametric analysis of K.

In the clinical study, generalized linear mixed model (GLMM) with a logit link function was used to analyze group differences in recovery over time, and outcome variables were dichotomized as “recovered” or “not recovered.” For group differences over time, *p*<0.05 was considered significant, but for pairwise comparisons at individual timepoints, *p*<0.01 defined significance to reduce the risk of type I error. GENSTAT V18 (VSNi International Ltd.) was used to perform GLMM and IBM SPSS Statistics for Windows (Version 25.0. Armonk, NY: IBM Corp) and was used to perform all other statistical analyses.

The sample size of 68 for the language validation was estimated on detecting a 10% difference between English and Japanese values for the word generation task, with a two-tailed, matched, paired *t* test design, with the alpha error set at 0.05 and power at 0.9, with an estimated score of 10 words generated for the English version, and standard deviation of 2.5 words. The word generation task was chosen as it has the highest score and greatest variability of the 4 tests, and a 10% difference between language versions was used to define a clinically meaningful difference.

## Results

During the period from May until August 2018, 68 volunteers participated in the initial validation study. They completed all four timepoints in both English and Japanese languages. The demographic data for volunteers are shown in Table 1.

**Table 1:** Demographic data of Japanese-English bilingual volunteers living in Australia

Characteristics	Volunteers (***n*** = 68)
Age, median (IQR), years	43.0 (18-68)
Female, no. (%)	53 (77.9)
BMI, median (IQR), kg/m^2^	20.3 (17.5-23.1)
Education, median (IQR), years	16.5 (12.5-20.5)
Years in Australia, median (IQR), years	13.0 (0-33.0)

*BMI* body mass index (kg/m^2^)

**Fig. 1:**
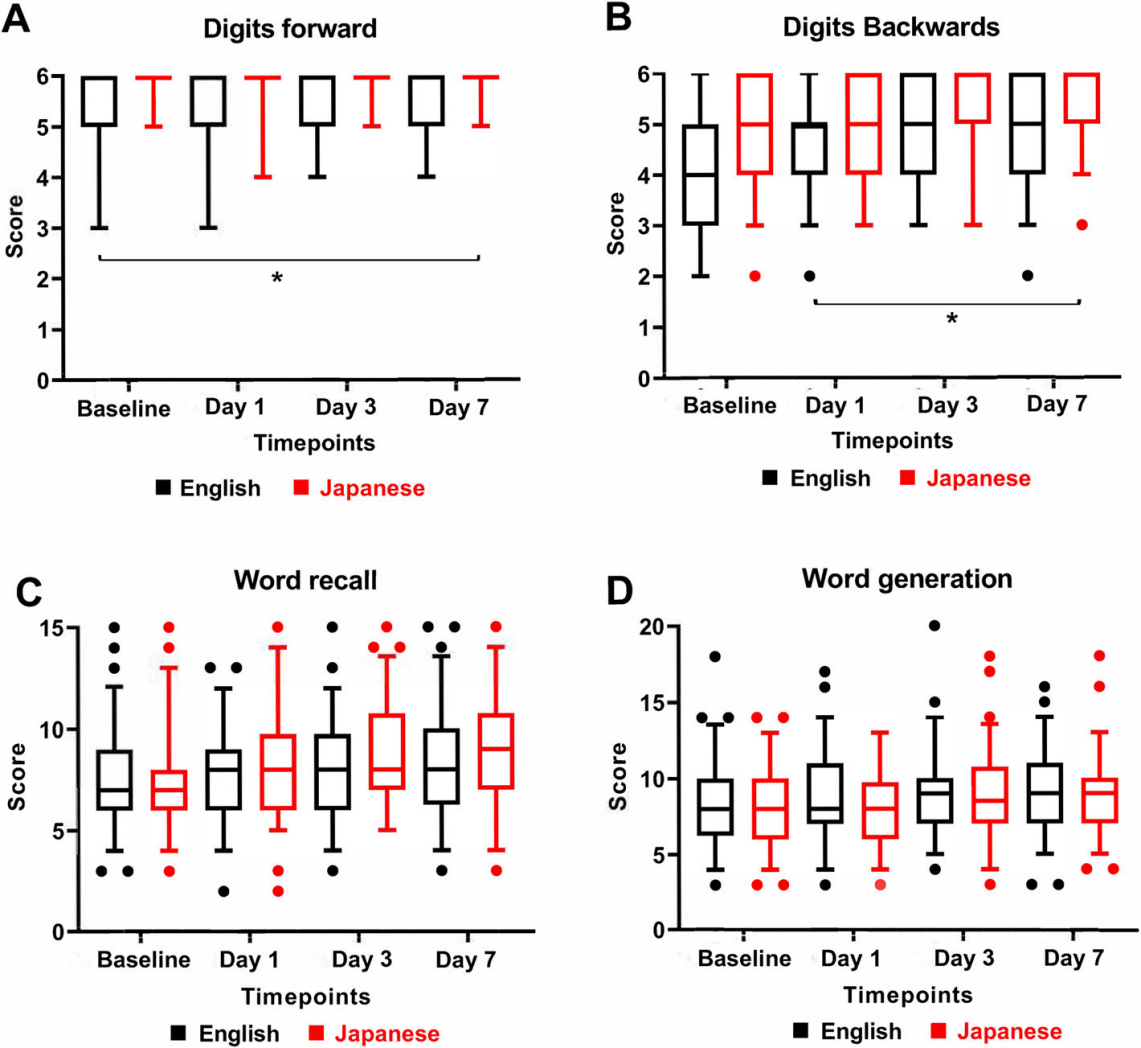
Cognitive recovery of the Postoperative Quality of Recovery Scale within bilingual volunteers. **a** Digit forwards, **b** digits backward, **c** word recall, and **d** word generation within the Japanese-English bilingual volunteers across each timepoints of the study. The graphs are displayed as a box and whisker plot with the line being the median, the box showing the interquartile range and the whiskers showing the 5 and 95 percentiles of the data including outliers.*p<0.5 for pairwise comparisons using Kruskal-Wallis *H* test the non-parametric analysis of K

Both the Japanese and English versions were not different in recovery scores at any timepoints within the nociceptive, emotional, and ADL domains. The scores for the cognitive domain are shown in Fig. 1. The scores for digits forwards (Fig. 1a) and digits backwards (Fig. 1b) were significantly greater when the PostopQRS was completed in Japanese. However, for word recall (Fig. 1c) and word generation (Fig. 1d), there was no significant differences between the Japanese and English versions at any of the timepoints. The difference between scores for digits forwards and digits backwards was <10% between groups at all timepoints.

From August 2018 until January 2019, a total of 51 patients were enrolled into a study at Miyazaki University Hospital, to assess discriminant validity between two surgical cohorts (including 22 ENT and 29 orthopedic patients). The patient demographic data is shown in Table 2. No harms or unintended events occurred to any of the patients.

**Table 2:** Patient demographics at Miyazaki Hospital

Variable	ENT (***n***=22)	Orthopedic (***n***=29)
Age, median (IQR), years	58.7 (26.7-90.7)	66.7 (50.5-82.9)
Female, *n* (%)	14 (63.6)	23 (79.3)
BMI, median (IQR), kg/m^2^	22.6 (16.5-28.7)	25.8 (20.4-30.2)
ASA, *n* (%)		
1-2	19 (86.4)	26 (89.7)
3	3 (13.6)	3 (10.3)
Operation description	Intraoral surgery: 7 Nasal surgery: 4 Ear surgery: 3 Neck dissection: 3 Others: 5	THR: 17 TKR: 7 Others: 5
Anesthesia technique	GA: 22 (TIVA: 9, inhalation gas: 13))	CSEA: 21 Spinal: 7 GA + Epi: 1
Duration of anesthesia, mean (SD), min	140 (5-275)	160 (117-203)

*ASA* American Society of Anesthesiologists Classification score, *GA* general anesthesia, *BMI* body mass index (kg/m^2^), *CSEA* combined spinal and epidural anesthesia, *THR* total hip replacement, *TKR* total knee replacement, *TIVA* total intravenous anesthesia

**Fig. 2:**
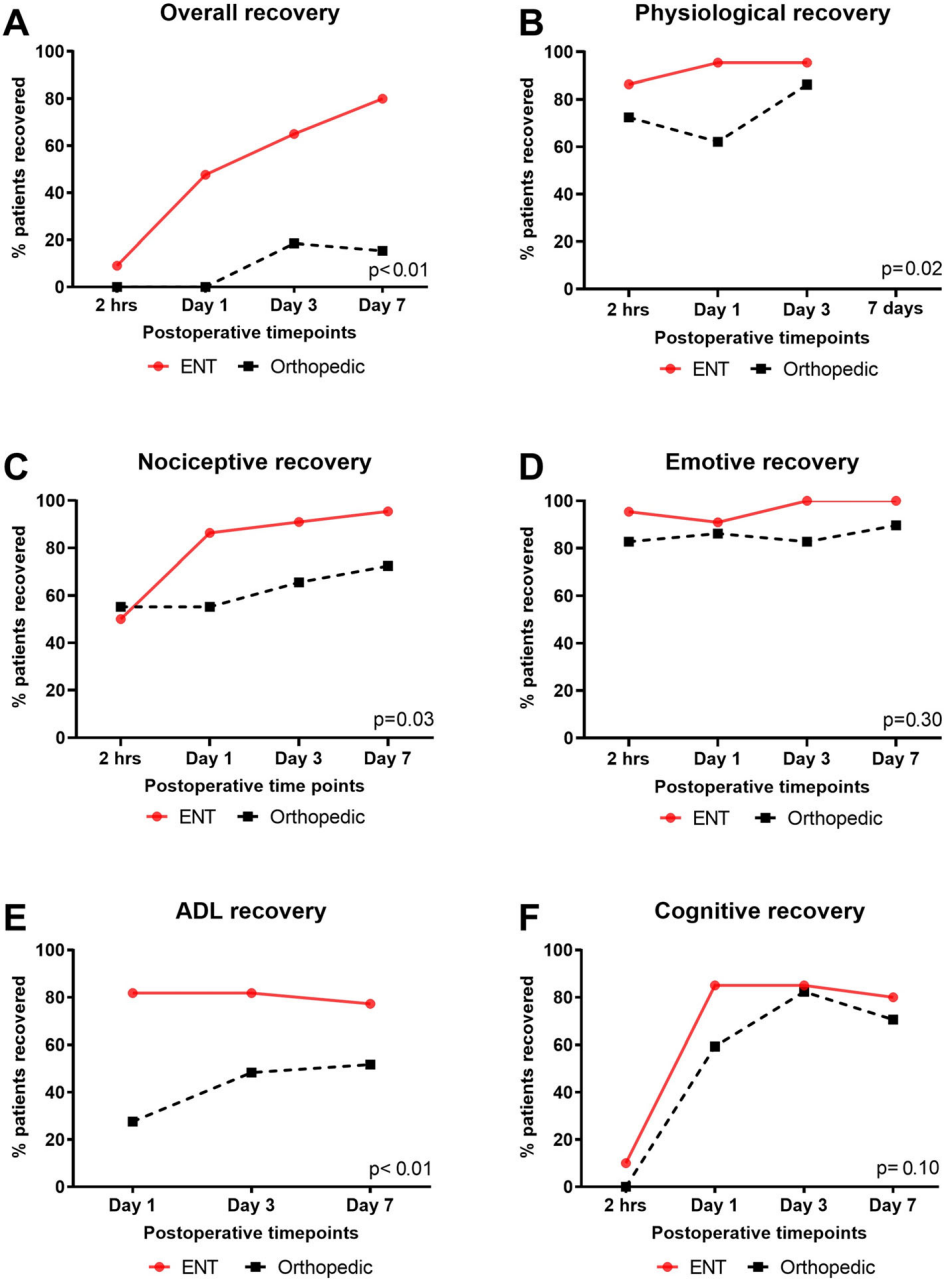
Individual domain recovery of the Postoperative Quality of Recovery Scale from the Clinical study. **a** Overall recovery (recovery within all individual domains of the PostopQRS) within the two surgical cohorts at Miyazaki University Hospital. **b** Physiological domain recovery, **c** nociceptive domain recovery, **d** emotive domain recovery, **e** ADL domain recovery, and **f** cognitive domain recovery. All *p* values for group differences over time are presented by the GLMM and represent time by treatment interactions

The recovery profiles for the recovery domains are shown in Fig. 2. Orthopedic patients had a significantly worse recovery profile in the ADL (*p*<0.01) and in overall recovery (*p*<0.01). There was a significant difference shown between the two groups in the physiological (*p*=0.02) and nociceptive (*p*=0.03) domains. The orthopedic group was worse than the ENT group at day 1 in the physiological domain (*p*<0.01).

**Fig. 3:**
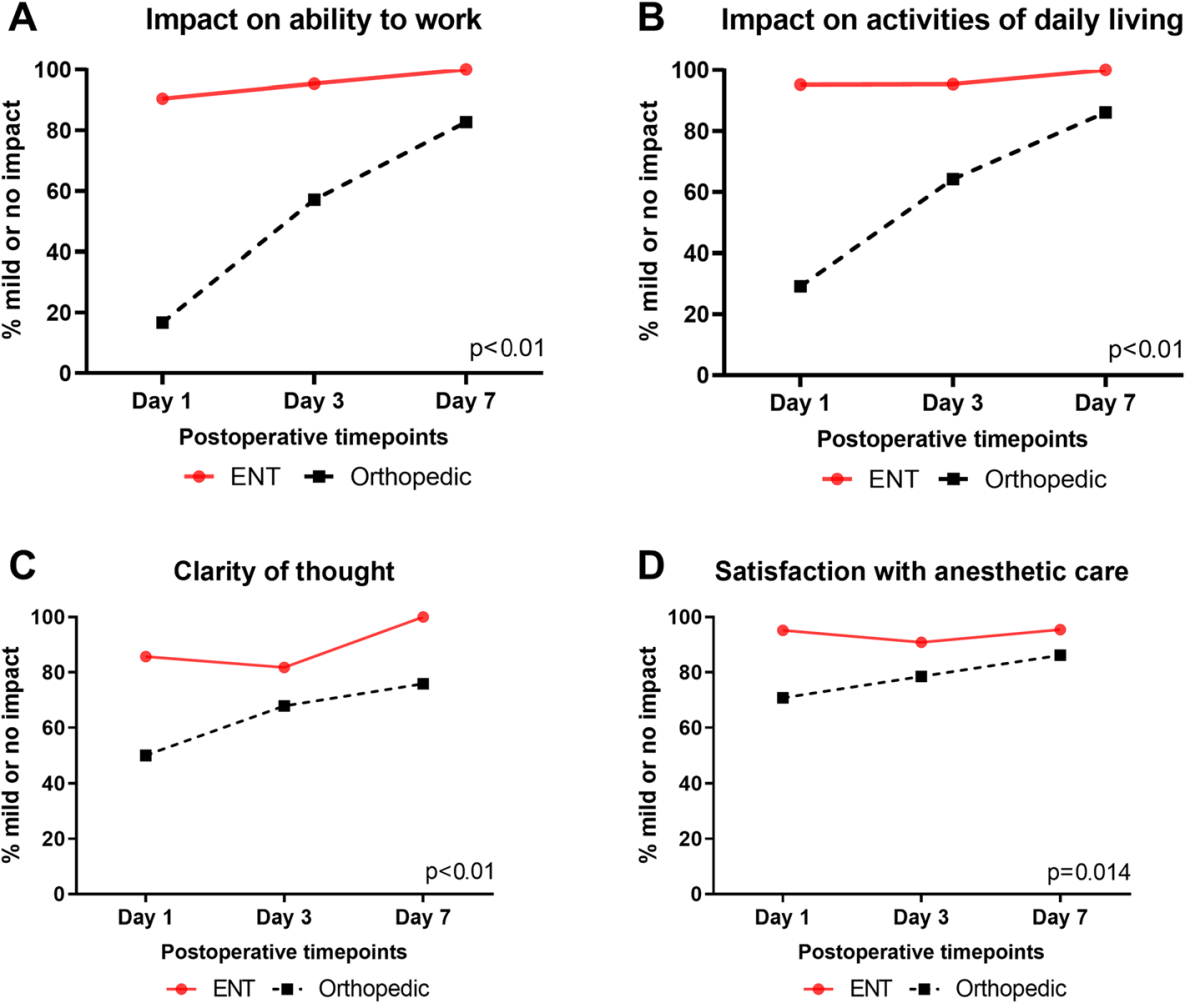
Overall patient perspective. Patient perspectives of the procedure included the operations impact on their ability to work, effect on their activities of daily living, clarity of thought, and overall satisfaction with the anesthetic care. All *p* values for group differences over time are presented by the GLMM and represent time by treatment interaction

The patient perspective domain is a subjective assessment from the patient on the impact of surgery, on their ability to work and to perform normal daily activities, clarity of thought, and overall satisfaction with anesthetic care, which was conducted from day 1. The change in overall patient perspective over time is shown in Fig. 3. For orthopedic patients, surgery and anesthesia had a greater impact on the ability to work (*p*<0.01), impact on ADL (*p*<0.01), and clarity of thought (*p*<0.01). Patients in the orthopedic group were less satisfied with the anesthetic care when compared to the ENT surgical cohort (*p*=0.014).

For understanding and satisfaction of the Japanese version of the PostopQRS, we asked additional questions and obtained responses from 28 patients. Most participants found the PostopQRS to be clear and easy to understand (85.7%), most were also satisfied with the PostopQRS (85.7%) and almost all participants responded positively to being evaluated with the PostopQRS again (92.9%), these questions are shown in Supplemental table 1. All 51 patients who participated in this study in the Japanese population cohort were able to complete the assessments for all the timepoints that were included.

## Discussion

This study shows that the English and Japanese versions of the PostopQRS are similar, and that the Japanese version was also able to discriminate recovery profiles between ENT and orthopedic patients. Feasibility and acceptability were also shown in the Japanese PostopQRS.

In previous studies, the PostopQRS has been successfully translated into other languages and validated in each of the countries [2, 8–10]. The cognitive domain is of great importance in the validation of the translation form. In particular, the word generation task is sensitive to language and cultural differences. In the English version, the task is to generate as many words as possible in 30 s using a defined letter of the alphabet (e.g., C). Languages that are not alphabet based, such as Chinese or Japanese, require a different set of sounds or characters to substitute for letters. In the Chinese version, substitution of a morpheme at the start of a word produced lower score values than the English version. However, this was corrected when the morpheme could be used as any part of the word [8]. In the Japanese language, there is cursive syllabary called “Hiragana” which has similarities to the English alphabet. Each character has a sound made up of a combination of a vowel and a consonant that can be written in English, and a word is made together with other Hiragana. In this study, we used the Hiragana characters; “う(u),” “か (ka),” and “せ (se),” used as the first part of the word, equivalent to the English version. We tested for similarity between languages using an arbitrary definition of <10% difference between scores in the cognitive domain, as well as assessing for statistical difference. Two of the cognitive tasks (digits forwards and digits backwards) were significantly different (favoring Japanese version), but the absolute difference was <10%. The cohort of volunteers was all Japanese native speakers and mostly tertiary educated. The scores were uniformly high for these tasks, leading to a skewed data set and a ceiling effect, with very low variability in the Japanese version. Taken together, these effects led to a limitation where the data were statistically different, even though the magnitude of the difference was small.

For the clinical study, we chose two surgery types which are known to have different recovery profiles, with different subdomains contributing to failed recovery [4]. Our findings are consistent with other studies that have shown improved recovery profiles over time, major joint replacement procedures take a longer time for patients to return to a recovered state when comparing it to other procedures [13, 14]. The major contributors to failed recovery in the orthopedic group were nociception (pain) and ADL. The patient reported impact is consistent with the differences between surgery types, including the impact on daily living, work, and satisfaction. Satisfaction is closely correlated with pain, more so than other aspects of the quality of recovery [1, 15].

We also investigated the feasibility and acceptability of the Japanese version of the PostopQRS. In previous studies [2, 10], the time taken to perform the assessments were around 5 min, consistent with the Japanese study. Acceptability was similar in the Japanese population to an Arabic population [9].

There are several limitations in this study. For the human volunteer study, by design of using bilingual speakers, we were likely to test a more educated stratum of society. Further, the sample may have been underpowered to demonstrate equivalence, but the data over time are similar between groups. Further, there could be inherent difference as the ability in each language may differ within individuals, as the birth language is likely to be stronger than the second language. Our sample size was based on only one of the tests in the cognitive battery which has data that is closest to continuous rather than categorical data, which may not be the best approach for all of the tests. Further, the definition of 10% difference to define a clinically meaningful difference is subjective but was based on prior language validation studies [8], and we kept that definition to improve consistency across language validations. However, other differences such as a 0.5-point difference may have been a better metric across the full battery of tests. Our clinical study was small and only tested surgery types where known differences of recovery were likely to occur. As an observational study, we did not control for the type of anesthesia or postoperative management, which could increase the heterogeneity of the recovery profiles. Attrition was low, but it is possible that patients refusing to participate in the study could represent a worse recovery group. Future studies are required in the Japanese population to benchmark recovery in many types of surgery and patient cohorts. The samples size for this study was too small to allow subgroup analysis.

## Conclusion

The Japanese version of the PostopQRS is similar to the English version for assessment of cognition and other domains. The assessment tool was able to show discriminant validity as well as feasibility and acceptability.

## Supplementary Information

**Additional file 1: Supplemental file 1.** English version of the PostopQRS.

**Additional file 2: Supplemental file 2.** Japanese version of the PostopQRS.

**Additional file 3: Supplemental table 1.** Additional questions to test acceptability of the PostopQRS.

## Data Availability

The dataset supporting the conclusion of this article will be made available upon request. It is currently stored in the repository of the Postoperative Quality of Recovery Scale website, postopqrs.com.

## Abbreviations

ADL: Activities of daily living
ANOVA: Analysis of variance
ENT: Ear-nose-throat
GLMM: Generalized linear mixed model
PostopQRS: Postoperative Quality of Recovery Scale
THR: Total hip replacement
TKR: Total knee replacement
