# Supplementary material for: The validation of a Japanese language version of the postoperative quality of recovery scale: a prospective observational study
Source: JA Clin Rep. 2021 Apr 9;7:32. doi: 10.1186/s40981-021-00432-0 (PMC8035378; doi:10.1186/s40981-021-00432-0)
Supplement: Supplementary file 1 — Additional file 1: Supplemental file 1. English version of the PostopQRS. [file 40981_2021_432_MOESM1_ESM.pdf]

Supplemental File 1. English language version of the PostopQRS

Baseline - Pre-surgery (baseline)

Nociceptive Factors

N1	**I am going to show you a series of faces and I would like you to indicate which face, number or description most accurately describes your level of pain at the moment?**<br>Please show the appropriate face chart to the patient and record the number corresponding to the actual response	☐ 1 = No pain<br>☐ 2 = Mild pain<br>☐ 3 = Moderate pain<br>☐ 4 = Severe pain<br>☐ 5 = Worst possible pain
N2	**I am going to show you a series of faces and I would you to indicate which face, number or description most accurately describes your level of feeling nauseous or vomiting at the moment?**<br>Please show the appropriate face chart to the patient and record the number corresponding to the actual response	☐ 1 = No nausea, dry retching or vomiting<br>☐ 2 = Mild nausea and no dry retching / vomiting<br>☐ 3 = Moderate nausea and or dry retching / vomiting<br>☐ 4 = Severe nausea and or dry retching / vomiting<br>☐ 5 = Continuous dry retching / vomiting

Emotional Factors

E1	**I am going to show you a series of faces and I would like you to indicate which face, number or description most accurately describes to what extent you feel sad, low or depressed at the moment?**<br>Please show the appropriate face chart to the patient and record the number corresponding to the actual response	☐ 1 = Not at all depressed / sad<br>☐ 2 = A little depressed / sad<br>☐ 3 = Somewhat depressed / sad<br>☐ 4 = Quite depressed / sad<br>☐ 5 = Extremely depressed / sad
E2	**I am going to show you a series of faces and I would like you to indicate which face, number or description most accurately describes to what extant you feel anxious or nervous at the moment?**<br>Please show the appropriate face chart to the patient and record the number corresponding to the actual response	☐ 1 = Not at all anxious / nervous<br>☐ 2 = A little anxious / nervous<br>☐ 3 = Somewhat anxious / nervous<br>☐ 4 = Quite anxious / nervous<br>☐ 5 = Extremely anxious / nervous

ADL Factors

A1	**Are you able to stand without assistance?**<br>Please record the number corresponding to the actual assessment	1 = Not at all<br>2 = With difficulty<br>3 = Easily
A2	**Are you able to walk without assistance?**<br>Please record the number corresponding to the actual assessment	1 = Not at all<br>2 = With difficulty<br>3 = Easily
A3	**Are you able to eat or drink without assistance?**<br>Please record the number corresponding to the actual assessment	1 = Not at all<br>2 = With difficulty<br>3 = Easily
A4	**Are you able to dress yourself without assistance?**<br>Please record the number corresponding to the actual assessment	1 = Not at all<br>2 = With difficulty<br>3 = Easily

Cognitive Factors

C1	**Please tell me your name, the city we are in and your date of birth.**<br>Please record the number of correct responses	# Correct responses
C2	**I am going to read you a list of numbers. Listen carefully, then when I am finished, I would like you to repeat them back to me in the same order that I read them. So, for example, if I said 1,2,3, you would say 1,2,3.**<br>Read out the digits given at the rate of one per second. Stop after failure at any point. Please record the item number of the last line correctly recalled.	5, 6 — 1 =<br>1, 6, 4 — 2 =<br>7, 1, 9, 4 — 3 =<br>8, 3, 9, 6, 2 — 4 =<br>5, 2, 8, 7, 9, 4 — 5 =<br>6, 8, 5, 1, 3, 9, 7 — 6 =
C3	**I am going to read you some more numbers, but this time when I stop I would like you to say them in reverse order. So, for example, if I said 1,2,3 you would say 3,2,1.**<br>Read out the digits given at the rate of one per second. Stop after failure at any point. Please record the item number of the last line correctly recalled.	3, 4 — 1 =<br>1, 5 ,9 — 2 =<br>6, 2, 7, 3 — 3 =<br>8, 4, 7, 6, 1 — 4 =<br>9, 2, 4, 7, 1, 3 — 5 =<br>4, 1, 6, 9, 5, 2, 7 — 6 =
C4	**I am going to read out a list of words. Please listen carefully as when I have finished I would like you to repeat back to me as many of the words as you can remember. You can say them in any order and if you are not sure if you have said a word, say it just in case.**<br>Read the words to the patient at about 1 per second. Please record the number of correct responses	**DESK, RANGER, BIRD, SHOVEL, STOVE, MOUNTAIN, GLASSES, TOWEL, CLOUD, BOAT, LAMB, GUN, PENCIL, CHURCH, FISH,**<br># Correct responses

C5	**I am going to name a letter and I would like you to state as many words as you can in 30 secs that begin with this letter, try to avoid proper nouns, such as peoples names, names of countries etc, numbers or the same word with a different ending such as long, longer, longish. The letter is "F".**<br>Time for 30 seconds using a stopwatch and stop patient at this time point. Please record the number of words correctly given in the 30 second time period.	# Correct responses ☐
