# Supplementary material for: The validation of a Japanese language version of the postoperative quality of recovery scale: a prospective observational study
Source: JA Clin Rep. 2021 Apr 9;7:32. doi: 10.1186/s40981-021-00432-0 (PMC8035378; doi:10.1186/s40981-021-00432-0)
Supplement: Supplementary file 2 — Additional file 2: Supplemental file 2. Japanese version of the PostopQRS. [file 40981_2021_432_MOESM2_ESM.pdf]

Supplemental File 2. Japanese version of the PostopQRS

Japanese version of the baseline Postoperative Quality of Recovery Scale survey.

侵害刺激因子
N1 **今のあなたの痛みのレベルを教えてください。**<br>顔のチャートを患者に示し、実際の反応に応じた番号を記録してください。(1-5)
N2 **今のあなたの吐き気や嘔吐のレベルを教えてください。**<br>顔のチャートを患者に示し、実際の反応に応じた番号を記録してください。(1-5)

感情の因子
E1 **あなたが今感じている悲しみ、つらさ、落ち込みを表している、顔、数字、説明を示してください。**<br>顔のチャートを患者に示し、実際の反応に応じた番号を記録してください。(1-5)
E2 **あなたが今感じている不安、緊張感を表している、顔、数字、説明を示してください。**<br>顔のチャートを患者に示し、実際の反応に応じた番号を記録してください。(1-5)

日常生活活動(ADL)
A1 **あなたは立つことができますか？**<br>１．簡単に　２．なんとか ３．できない
A2 **あなたは歩くことができますか？**<br>１．簡単に　２．なんとか　３．できない
A3 **あなたは自分で食べたり飲んだりすることができますか？**<br>１．簡単に　２．なんとか　３．できない
A4 **あなたは自分で服を着られますか？**<br>１．簡単に　２．なんとか　３．できない

認知の因子
C1 **私にあなたの名前、今いる町の名前、あなたの生年月日を教えてください。**<br>正しく答えられた数を記載してください。(1-3)
C2 **私はあなたに一連の数字を読み上げます。注意深く聞いてください。それから、私が読み終えたら、私が読んだ順序で、再び言い返してください。たとえば、私が 1,2,3 と読んだら、あなたは 1,2,3 と繰り返してください。**<br>与えられた数字を１秒間に一つの割合で読み上げてください。失敗した時点で必ず止めてください。正確に思い出せた最終ラインの数字を記録してください。<br><br>1　5, 6<br>2　1, 6, 4<br>3　7, 1, 9, 4<br>4　8, 3, 9, 6, 2<br>5　5, 2, 8, 7, 9, 4<br>6　6, 8, 5, 1, 3, 9, 7
C3 **私はあなたにさらに数字を読み上げます。しかしこんどは、私が読むのをやめたら、反対の順序で言ってください。たとえば、私が 1,2,3 と読んだら、あなたは 3,2,1 と繰り返してください。**<br>与えられた数字を１秒間に一つの割合で読み上げてください。失敗した時点で必ず止めてください。正確に思い出せた最終ラインの数字を記録してください。<br><br>1　3, 4<br>2　1, 5, 9<br>3　6, 2, 7, 3<br>4　8, 4, 7, 6, 1<br>5　9, 2, 4, 7, 1, 3<br>6　4, 1, 6, 9, 5, 2, 7
C4 **私はあなたに一連の言葉を読み上げます。注意深く聞いてください。それから私が読み終えたら、**

**あなたが覚えているだけ多くの言葉を、私に繰り返してください。順番はなんでもかまいません。忘れたと思ったら、念のためもう一度言ってください。**<br>患者に１秒１単語の割合で読んでください。正答した数を記録してください。<br><br>「机」、「兵隊」、「鳥」、「シャベル」、「ストーブ」、「山」、「眼鏡」、「タオル」、「雲」、「ボート」、「羊」、「銃」、「鉛筆」、「教会」、「魚」
C5 **今から私は１文字を指定します。この文字で始まる言葉を 30 秒でできるだけたくさんあげてください。固有名詞、いわゆる人の名前や国の名前、また、数字や語尾が違うだけの同じ言葉**<br>**たとえば山、山なみ、山々などは避けてください。私が指定する文字は‘か’です。**<br>30 秒を計るのにストップ・ウォッチを使用してください。この時間がきたら患者をとめてください。30 秒の間に答えられた言葉の数を正確に記録してください。
