# Supplementary material for: The validation of a Japanese language version of the postoperative quality of recovery scale: a prospective observational study
Source: JA Clin Rep. 2021 Apr 9;7:32. doi: 10.1186/s40981-021-00432-0 (PMC8035378; doi:10.1186/s40981-021-00432-0)
Supplement: Supplementary file 3 — Additional file 3: Supplemental table 1. Additional questions to test acceptability of the PostopQRS. [file 40981_2021_432_MOESM3_ESM.pdf]

Supplemental Table 1. Additional Questions to test acceptability of the PostopQRS

Q1. Were questions clear and easy?	1= Not clear and easy at all<br>2= Somewhat clear and easy<br>3= Moderately clear and easy<br>4= Clear and easy<br>5= Totally clear and easy
Q2. Were you satisfied with the PostopQRS?	1= Not at all satisfied<br>2= Somewhat satisfied<br>3= Moderately satisfied<br>4= Satisfied<br>5= Totally satisfied
Q3. Do you want to be evaluated again with this questionnaire?	1= Not at all<br>2= Somewhat<br>3= Moderately<br>4= Very<br>5= Totally
